# Cyanocobalamin-Modified Colistin–Hyaluronan Conjugates: Synthesis and Bioactivity

**DOI:** 10.3390/ijms241411550

**Published:** 2023-07-17

**Authors:** Natallia V. Dubashynskaya, Anton N. Bokatyi, Tatiana S. Sall, Tatiana S. Egorova, Yuliya A. Nashchekina, Yaroslav A. Dubrovskii, Ekaterina A. Murashko, Elena N. Vlasova, Elena V. Demyanova, Yury A. Skorik

**Affiliations:** 1 Institute of Macromolecular Compounds of the Russian Academy of Sciences, Bolshoi VO 31, St. Petersburg 199004, Russia; 2 Institute of Experimental Medicine, Acad. Pavlov St. 12, St. Petersburg 197376, Russia; 3 State Research Institute of Highly Pure Biopreparations, Pudozhsakya 7, St. Petersburg 197110, Russia; 4 Institute of Cytology of the Russian Academy of Sciences, Tikhoretsky 4, St. Petersburg 194064, Russia; 5 Almazov National Medical Research Centre, Akkuratova 2, St. Petersburg 197341, Russia

**Keywords:** colistin, hyaluronic acid, cyanocobalamin, polymeric conjugates, oral drug delivery, intestinal permeability

## Abstract

Polymeric drug delivery systems enhance the biopharmaceutical properties of antibiotics by increasing their bioavailability, providing programmable and controlled-release properties, and reducing toxicity. In addition, drug delivery systems are a promising strategy to improve the intestinal permeability of various antimicrobial agents, including colistin (CT). This study describes the modification of conjugates based on CT and hyaluronic acid (HA) with cyanocobalamin (vitamin B12). Vitamin B12 was chosen as a targeting ligand because it has its own absorption pathway in the small intestine. The resulting polysaccharide conjugates contained 95 μg/mg vitamin B12 and the CT content was 335 μg/mg; they consisted of particles of two sizes, 98 and 702 nm, with a ζ-potential of approximately −25 mV. An in vitro release test at pH 7.4 and pH 5.2 showed an ultra-slow release of colistin of approximately 1% after 10 h. The modified B12 conjugates retained their antimicrobial activity at the level of pure CT (minimum inhibitory concentration was 2 μg/mL). The resulting delivery systems also reduced the nephrotoxicity of CT by 30–40% (HEK 293 cell line). In addition, the modification of B12 improved the intestinal permeability of CT, and the apparent permeability coefficient of HA–CT–B12 conjugates was 3.5 × 10^−6^ cm/s, corresponding to an in vivo intestinal absorption of 50–100%. Thus, vitamin-B12-modified conjugates based on CT and HA may be promising oral delivery systems with improved biopharmaceutical properties.

## 1. Introduction

Modification of known drugs is a simple and cost-effective strategy for pharmaceutical research and development, especially for antimicrobial drugs [1,2]. Bacterial resistance to modern antibiotics is increasing rapidly, and the development of new-generation antimicrobial agents is a long and expensive process; moreover, most synthesized molecules fail in the preclinical and clinical stages due to their poor biopharmaceutical properties [3]. Multidrug-resistant bacteria, including multidrug-resistant Gram-negative ESKAPE pathogens, exhibit resistance to three or more classes of antibiotics (e.g., carbapenems and third-generation cephalosporins) and are a major cause of mortality [4]. One potential antimicrobial drug that could benefit from various modifications of a known molecule is the peptide antibiotic colistin (CT) [5,6,7]. The disadvantages of CT therapy are its severe side effects such as nephrotoxicity and neurotoxicity [8,9,10]; moreover, CT is practically not absorbed in the gastrointestinal tract (GIT), which limits the possible routes of its administration. Various nanotechnology-based delivery systems could improve the oral administration of CT [11,12].

The oral route of drug administration is more popular, and it is a relatively convenient and safe method of drug administration; oral administration is associated with higher patient compliance. However, absorption through the intestinal barrier is a prerequisite for the pharmacological effect of oral drugs [13]. Important factors influencing drug absorption and intestinal permeability are pH, enzymes, mucus, and the unstirred water layer, as well as the gastrointestinal membrane barrier. The main mechanisms of drug absorption across the gastrointestinal membrane are paracellular and transcellular transport. The paracellular pathway through the aqueous pores between the epithelial cells is the main mechanism for absorption of hydrophilic charged molecules or ions with a typical molecular weight cut-off of 200–300 Da. In turn, transcellular transport can be realized by simple passive diffusion (relevant for small lipophilic molecules), carrier-mediated transport (active transport and facilitated diffusion), and endocytosis (the primary transport mechanism for macromolecules and particles larger than 500 nm) [14].

An example of a carrier-mediated active transport system in the small intestine, the ileum, includes the membrane transporters of cobalamin (vitamin B12). Absorption of vitamin B12 is a complex and highly efficient process involving the interaction of three transport proteins—haptocorrin, intrinsic factor, and transcobalamin II—and several receptors. Haptocorrin, also known as transcobalamin I, protects B12 from acid hydrolysis in gastric fluid and aids in the utilization of vitamin B12 analogues. Gastric intrinsic factor selectively binds dietary vitamin B12 and then enters intestinal cells via receptor-mediated endocytosis. Vitamin B12 taken up by the cells is converted to transcobalamin II and released into the systemic circulation [15,16]. As a result of its own transport system, vitamin B12 has been widely used as a targeting ligand to improve the intestinal permeability of drugs, including peptide molecules [17,18]. The strategy of improving the oral delivery of peptides and proteins by modification with vitamin B12 began with the basic research of the Russell-Jones scientific group in the 1990s, on the synthesis and oral delivery of granulocyte-colony-stimulating factor, luteinizing-hormone-releasing hormone, and erythropoietin with proven efficacy in vivo [19,20,21]. Currently, conjugation with vitamin B12 is being used to develop polymeric systems for oral insulin delivery. The use of B12-modified nanoparticles enhances uptake and intestinal permeability for efficient oral drug delivery, and also reduces drug toxicity [22].

Verma et al. [23] used vitamin B12 as a targeting ligand to prepare layered calcium phosphate nanoparticles for oral insulin delivery, where vitamin-B12-grafted chitosan (MW of 65–90 kDa, degree of deacetylation of 75–85%) and sodium alginate (15–20 cP, 1% in H_2_O) were the polycation and polyanion, respectively. Vitamin B12 was linked through the OH group of the ribose tail to the amino group of chitosan via a succinyl linker using carbodiimide chemistry. The developed nanoparticles had a size of approximately 250 nm and a ζ-potential of +32 mV. It was shown that vitamin B12, as a targeting ligand, significantly increased the bioavailability of insulin by oral administration through receptor uptake. In vitro experiments on the Caco-2 monolayer showed the higher absorption of nanoparticles based on vitamin-B12-modified chitosan compared to nanoparticles based on original chitosan. Furthermore, an in vivo study in diabetic Wistar rats showed a four-fold increase in insulin bioavailability and a sustained hypoglycemic effect within 12 h after administration of vitamin-B12-containing nanoparticles compared to nanoparticles without vitamin B12.

Previously, we synthesized CT conjugates with chitosan and hyaluronic acid (HA) for the parenteral delivery of CT. The resulting conjugates, with degrees of substitution (DS) of 3–10%, self-assembled in water into nanosized particles (from 100 to 600 nm) and were resistant to hydrolysis (in vitro CT release at pH 7.4 and pH 5.2 was 1–5% after 24 h). Furthermore, the degree of substitution with CT fragments of 8–10% provided antimicrobial activity at the level of pure CT, as well as reduced cytotoxicity [7,24].

The aim of the present study was to obtain vitamin-B12-modified conjugates of CT with HA and to investigate their potential in terms of the oral delivery of CT. It was hypothesized that functionalization with vitamin B12 would enhance intestinal permeability and absorption of the polymeric conjugates through receptor-mediated endocytosis. The concept of conjugating CT to polymers to improve its biopharmaceutical properties has been used previously [25,26,27,28]. However, to the best of our knowledge, this study was the first attempt to develop an oral form of CT that can be absorbed in the GI tract by grafting vitamin B12 as a targeting ligand.

## 2. Results and Discussion

### 2.1. Synthesis of the Succinyl Cyanocobalamin (Suc-B12)

**Figure 1:**
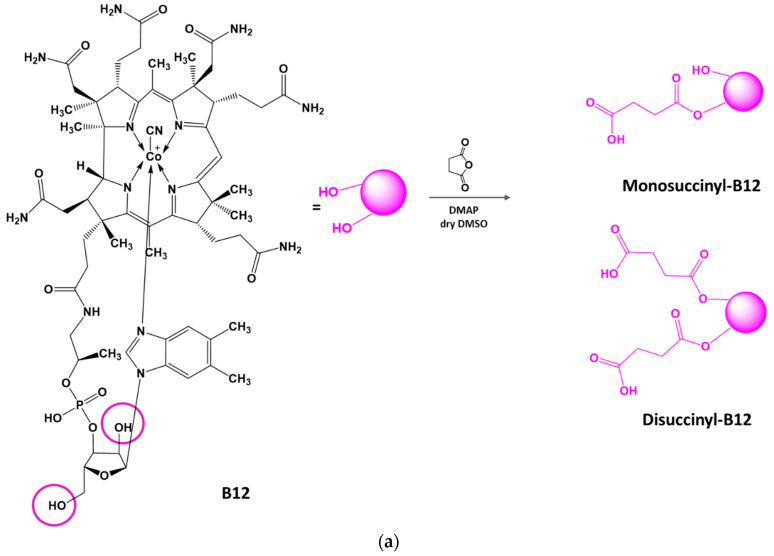

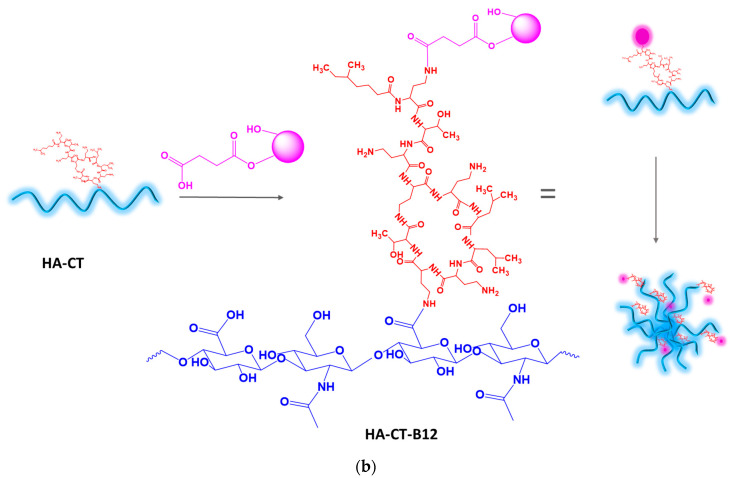
Schemes for the preparation of succinylated vitamin B12 (**a**) and the modification of HA–CT by Suc-B12 (**b**).

Analysis of the literature showed that, for the synthesis of succinyl derivatives of cyanocobalamin, the OH group of the ribose (substitution occurs first, resulting in monosubstituted succinyl-B12) or the less reactive capable secondary hydroxyl group (a significant excess of reagent results in disubstituted succinyl-B12) can be used (Figure 1a) [29]. The reaction is typically performed in dry DMSO under anhydrous conditions in the presence of DMAP or pyridine as electron donors [23,29,30,31]. This type of conjugation does not interfere with the recognition of vitamin B12 by the transfer proteins involved in its absorption [15].

**Figure A1:**
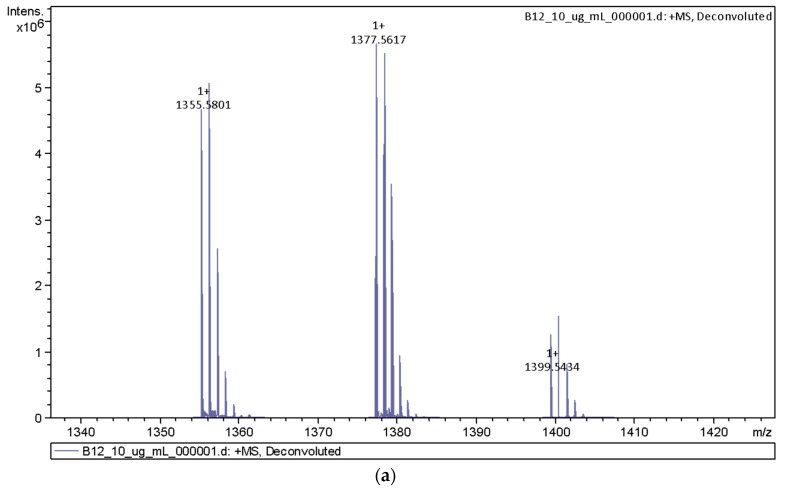

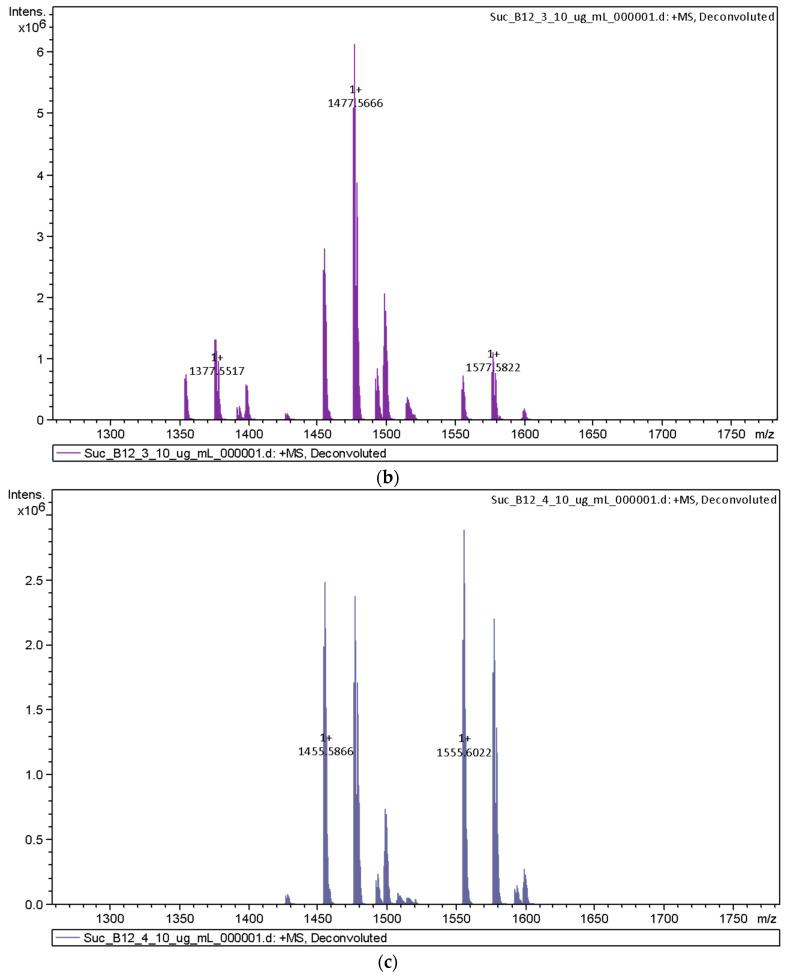
MS spectra of vitamin B12 (**a**), Suc-B12-10 (**b**), and Suc-B12-100 (**c**).

We used succinic anhydride in 10- and 100-fold molar excesses over vitamin B12, resulting in products (Suc-B12-10 and Suc-B12-100, respectively) that differed in composition according to the mass spectrometry data (Table 1, Figure A1). For example, we observed both B12 (1355), and sodium (1377) and disodium (1399) forms of B12 in the mass spectrum of the parent vitamin B12 (Figure A1a). The Suc-B12-10 sample contained both the parent B12 (22.4%) and B12 succinyl derivatives: 74.1% monosuccinyl B12 (1477) and 3.5% disuccinyl B12 (1577) (Table 1, Figure A1b). Compared to Suc-B12-10, Suc-B12-100 contained almost no parent B12 (0.4%), but more of both monosuccinyl B12 (87.8%) and disuccinyl B12 (11.8%) (Table 1, Figure A1c). For further modification of the HA–CT conjugate, we used the Suc-B12-100 sample.

### 2.2. Modification of the HA–CT Conjugate with Suc-B12

For B12 modification, we used previously synthesized HA–CT conjugates (CT content was 367 ± 1 μg/mg, the apparent hydrodynamic diameter (D_h_) was 50 and 560 nm, and the ζ-potential was −20 mV) [7]. A study of HA–CT conjugates showed that the resulting compound was sufficiently stable, with less than 3% CT released within 24 h at pH 7.4 [7].

To obtain modified HA–CT–B12 conjugates, we exploited the carbodiimide activation of the carboxyl groups of Suc-B12 and their further coupling to the amino groups of the conjugated CT (Figure 1b). We used Suc-B12 in an amount of 10 mol% (30% by mass) relative to HA–CT, because this amount is able to provide an acceptable level of intestinal permeability of nanoformulations according to [23]. According to spectrophotometry, the B12 content in the HA–CT–B12 conjugate was 95 μg/mg (Table 2).

**Figure 2:**
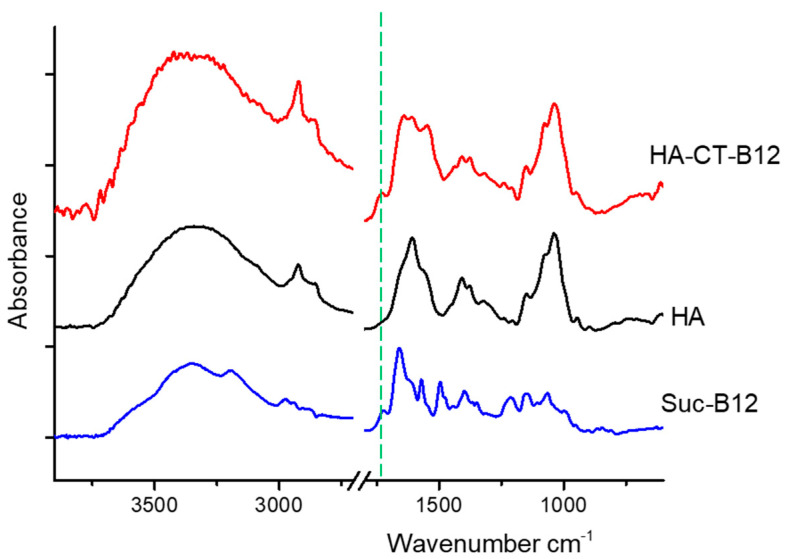
FTIR spectra of the Suc-B12-100, HA, and HA–CT–B12.

The coupling of vitamin B12 to the HA–CT conjugate was demonstrated by Fourier transform infrared (FTIR) spectroscopy (Figure 2). In the FTIR spectrum of the HA–CT–B12 conjugate, a characteristic band was observed at 1730 cm^−1^, corresponding to the C=O vibrations of the ester bond of the succinyl linker of vitamin B12.

According to the dynamic light scattering (DLS) data, D_h_ of the HA–CT–B12 conjugate was 98 ± 22 nm, with the presence of some aggregates of 702 ± 248 nm (Table 2). This pattern was in good agreement with our previous DLS studies, in which the fast mode of the conjugates belonged to individual macromolecules (unimers) and the slow mode belonged to their aggregates. The ζ-potential of the conjugates was −25.4 ± 0.3 mV, indicating their colloidal stability (Table 2).

**Figure 3:**
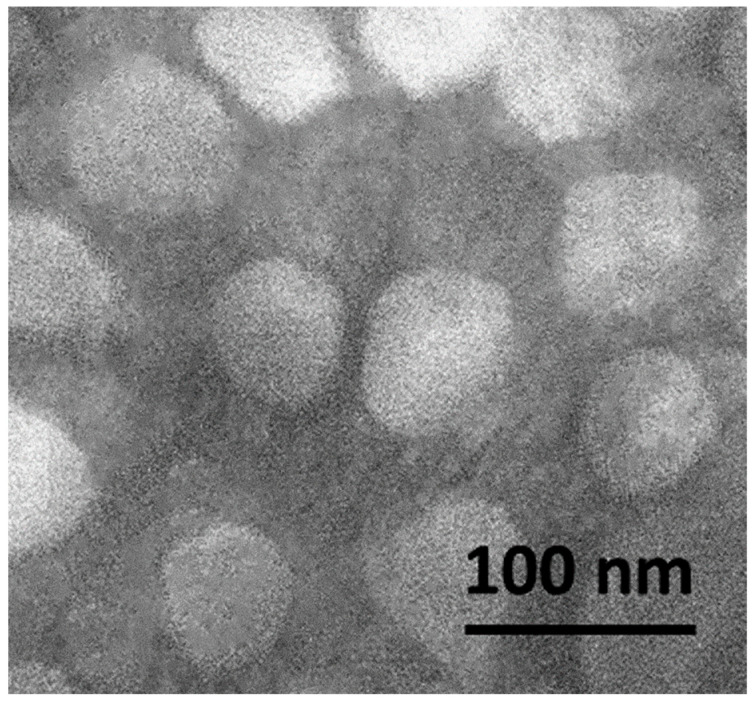
SEM image of HA–CT–B12 conjugate.

Scanning electron microscopy (SEM) confirmed the presence of spherical particles less than 100 nm in size, which was in good agreement with the DLS data (Figure 3).

### 2.3. The pH Stability Studies and In Vitro CT-Release Profile

Vitamin B12 is absorbed by active transport in the small intestine (i.e., in the ileum, with an average pH of 7.4–7.5) [32]. At the same time, the transit of the drug through different parts of the gastrointestinal tract with different pH levels and enzyme compositions affects its effectiveness; for successful absorption in the small intestine, the drug must be stable at the pH of the stomach (pH in the fasted/fed state) and reach the site of absorption as quickly as possible (to transit from the stomach to the intestine). Residence time in the stomach is typically from 5 min to 2 h; usually a meal prolongs the stay of the dosage form in the stomach. To accelerate the transit of the drug from the stomach to the small intestine, it is recommended to take it with water and on an empty stomach (in this case the drug almost immediately reaches the small intestine). The drug remains in the small intestine for an average of 3–4 h, independent of meals [14]. We sequentially studied the stability of the HA–CT–B12 conjugate at pH 1, 6.8, and 7.4 and showed that the synthesized compounds remained stable during transit through different parts of the gastrointestinal tract and were able to reach the target site (ileum) unchanged (Table 3). In turn, the stability at the pH of the ileum provided a good potential for the successful absorption of the conjugates into the blood.

**Figure 4:**
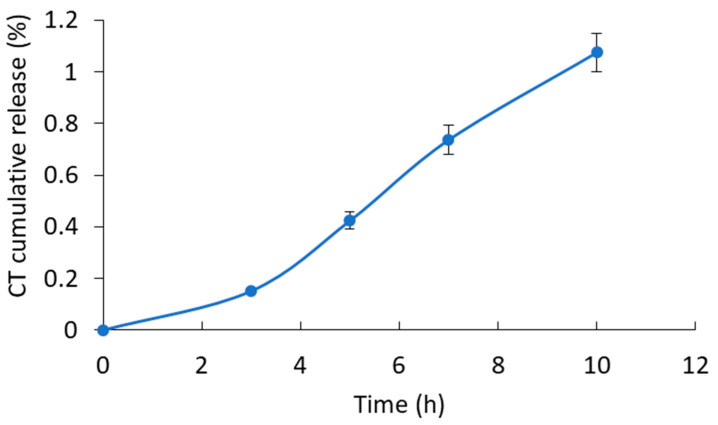
In vitro kinetics of CT release at 37 °C from the HA–CT–B12 conjugate in phosphate buffer at pH 5.2. Each point represents the mean of triplicate measurements ± SD (*n* = 3).

In vitro release kinetics studies of the designed conjugates at the inflammatory site pH (5.2) showed an ultra-slow release of CT via the hydrolysis of amide bonds (Figure 4), which was in agreement with our previous studies [7,24]. In summary, approximately 1% of CT was released in 10 h, and these slow kinetics require confirmation of the antimicrobial activity of CT in vitamin-B12-modified conjugates.

### 2.4. Antimicrobial Activity

Previously, we observed that conjugation of CT with HA at a DS of 8% did not reduce its potency compared to pure CT [7].

**Figure 5:**
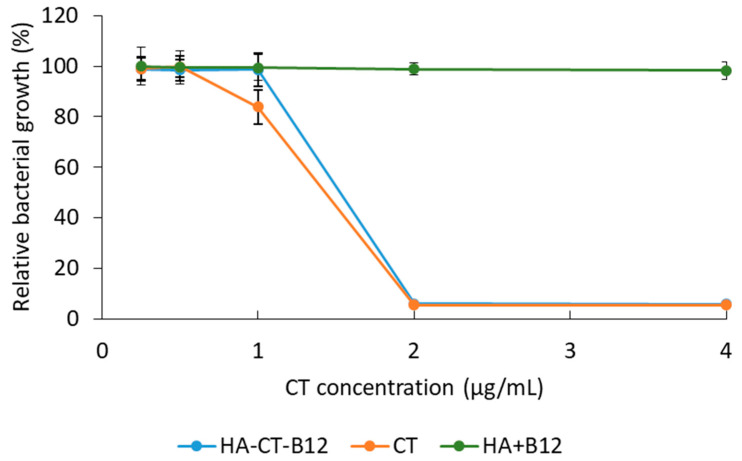
Minimum inhibitory concentrations (MICs) of HA–CT–B12 conjugates and CT against *P. aeruginosa*. Each point represents the mean of triplicate measurements ± SD (*n* = 3).

A study of antimicrobial activity against *Pseudomonas aeruginosa* (1 × 10^7^ CFU/mL) showed that the HA–CT–B12 conjugate also had antimicrobial activity comparable to that of pure CT. Both minimum inhibitory concentrations (MICs) were 2 μg/mL, indicating that the antibiotic activity was maintained despite the modification with vitamin B12. At the same time, a mixture of HA with vitamin B12 at an equivalent concentration had no significant effect on visible bacterial growth (Figure 5).

### 2.5. Cytotoxicity Study

**Figure 6:**
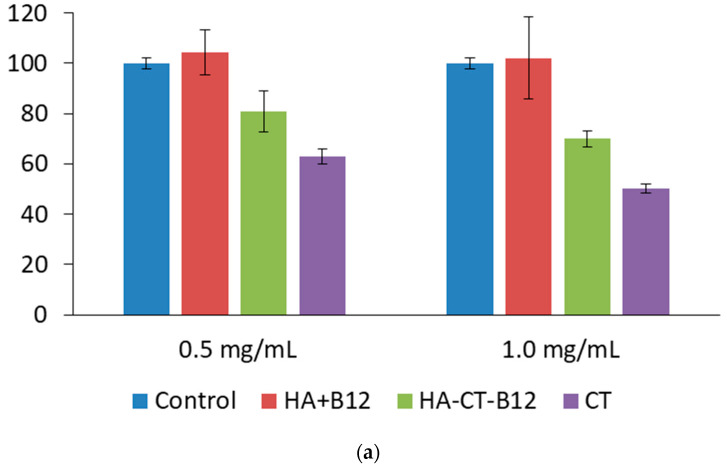

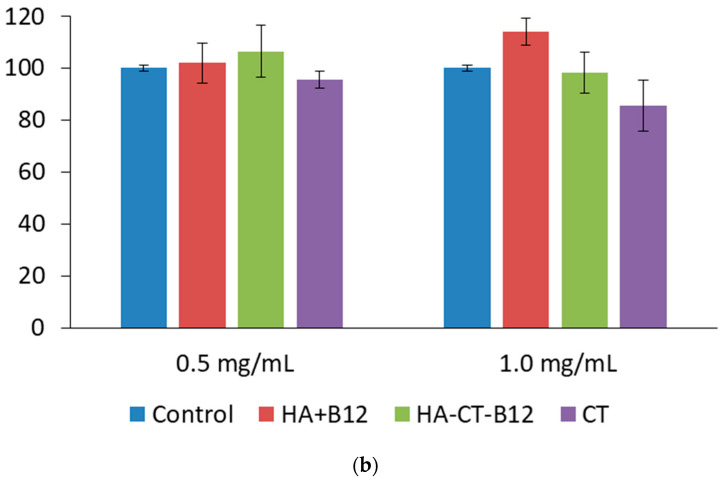
Viability of HEK 293 (**a**) and T 98G (**b**) cells incubated in the presence of HA–CT–B12 conjugate, HA+B12 mixture, and free CT for 72 h. The CT concentrations in the tested samples were 0.5 and 1.0 mg/mL. Data are expressed as mean ± SD (*n* = 5).

We investigated the potential nephro- and neurotoxicity of the new HA–CT–B12 conjugates on kidney (HEK 293) and brain (T 98G) cell lines. Equal amounts of HA+B12 mixture and free CT were used as controls. In vitro cytotoxicity experiments showed that the HA–CT–B12 conjugate increased cell viability by 1.3- and 1.4-fold compared to native CT at CT concentrations of 0.5 and 1.0 mg/mL, respectively (Figure 6a). At the same time, the concentrations of free CT tested had no toxic effect on glioblastoma cells; however, even in this experiment, the viability of T 98G cells in the presence of the HA–CT–B12 conjugate was approximately 10–15% higher than in the presence of native CT (Figure 6b). Thus, HA–CT–B12 conjugates reduced the toxicity of CT against kidney (HEK 293) and brain (T 98G) cells.

### 2.6. Caco-2 Cell Permeability Assay

The Caco-2 cell line (human colon adenocarcinoma) has enterocyte characteristics. After monolayer formation, the cells form junctional complexes and microvilli on the apical surface. It is known that the permeability of the Cao-2 cell monolayer is highly correlated with the processes of drug absorption in the intestine. The disadvantage of the Caco-2 cell line is its cancer origin and phenotypic instability; however, Caco-2 cells are widely used in pharmacological studies because they allow robust results to be obtained [29]. The apparent permeability coefficient in vitro (Papp) for substances absorbed through the intestinal wall by active transport had lower values compared to in vivo experiments because of the lower degree of expression of ionic and peptide transport proteins in Сасo-2 cells. Therefore, the main area of application of Сасo-2 cell culture is the qualitative assessment of intestinal permeability [33].

**Figure 7:**
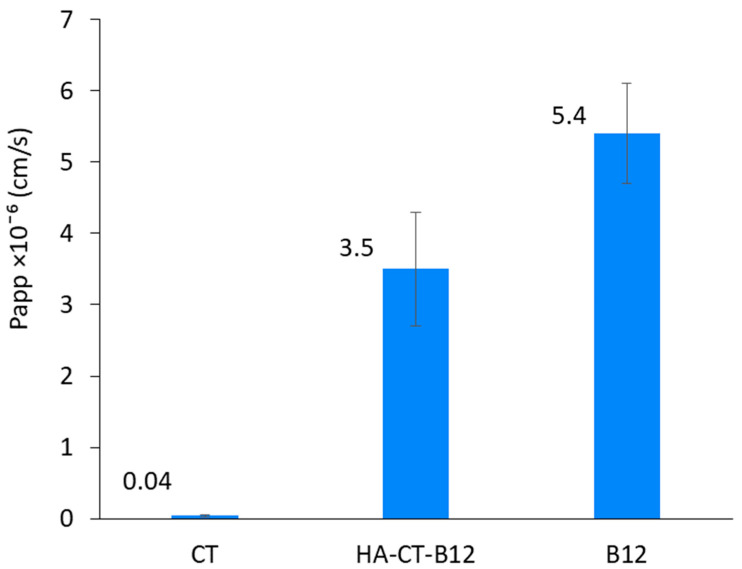
Apparent permeability coefficient of HA–CT–B12 conjugate, free B12, and free CT for 2 h. Data are presented as mean ± SD (*n* = 3).

In general, absorption in the human GIT is 50 to 100% for compounds with an in vitro Papp greater than 1 × 10^−6^ cm/s. These compounds are promising for the development of oral dosage forms [34,35]. Intestinal permeability testing in the Caco-2 cell model showed that the B12-modified conjugates based on CT and HA had an acceptable Papp to allow potential absorption in the gastrointestinal tract (3.5 × 10^−6^ cm/s). In comparison, the Papp of free CT and free vitamin B12 in this experiment were 0.04 × 10^−6^ cm/s and 5.4 × 10^−6^ cm/s, respectively (Figure 7).

## 3. Materials and Methods

### 3.1. Materials and Reagents

In this study, we used previously synthesized CT conjugates based on sodium hyaluronate (HA) with a DS for CT moieties of 8 mol% (HA–CT). The CT content in the HA–CT conjugates was 367 ± 1 μg/mg. In phosphate-buffered saline (PBS, pH 7.4), HA–CT conjugates were present as unimers and nanoparticles with hydrodynamic diameters (D_h_) of 50 and 560 nm, respectively; the ζ-potential was −20 mV. The HA (MW of 1.8 × 10^5^) was obtained from Shandong Focuschem Biotech (Qufu, Shandong, China); the D_h_ and ζ-potential in the PBS were 34 nm and −26 mV, respectively [7].

CT sulfate (the contents of CT A (polymyxin E1) and CT B (polymyxin E2) in the purchased product were 31.1 ± 0.4% and 68.9 ± 0.4% by liquid chromatography–mass spectrometry (LC–MS), respectively), cyanocobalamin (vitamin B12), 1-ethyl-3-(3-dimethylaminopropyl) carbodiimide hydrochloride (EDC), N-hydroxysuccinimide (NHS), dimethylaminopyridine (DMAP), succinic anhydride (SA), dimethyl sulfoxide (DMSO), PBS, disodium phosphate (Na_2_HPO_4_), monopotassium phosphate (KH_2_PO_4_), and sodium chloride (NaCl) were purchased from Sigma-Aldrich Co. (St. Louis, MO, USA).

DMEM (Dulbecco’s modified Eagle medium), penicillin/streptomycin, L-glutamine, trypsin, versine, and PBS were purchased from Biolot (St. Petersburg, Russia), and fetal bovine serum (FBS) was purchased from HyClone Laboratories (Logan, UT, USA).

### 3.2. Synthesis of Succinyl Cyanocobalamin (Suc-B12)

Suc-B12 was prepared according to the method described in [23] with certain modifications: B12 (0.05 g, 0.037 mM) was dissolved in 5 mL of dry DMSO, then an equimolar amount of DMAP and a 10- or 100-fold molar excess of succinic anhydride were added. The reaction mixtures were stirred for 24 h and the resulting products (Suc-B12-10 and Suc-B12-100, respectively) were precipitated with acetone and dried at 40 °C for one day.

The products were characterized by mass spectrometry using a maXis impact Q-TOF mass spectrometer (Bruker Daltonics GmbH, Bremen, Germany) equipped with an electrospray ionization (ESI) source (Bruker Daltonics GmbH, Bremen, Germany) operated in the positive ionization mode. Mass calibration was performed with sodium formate solution (calibration mode HPC, standard deviation (SD) 0.308 ppm). Flow injection mode was used for analysis: mass range from 50 to 1300 *m*/*z*, spectra rate 1 Hz, and line and profile spectra stored. The acquisition parameters were as follows: Source: end plate offset 500 V, capillary 4500 V, nebulizer 0.4 bar, dry gas 4.0 L/min, and dry temperature 180 °C. Collision cell: collision energy 7.0 eV, transfer time 90.0 μs, and pre-pulse storage 10.0 μs. Mass spectra were analyzed and deconvoluted using DataAnalysis^®^ 5.0 software (Bruker Daltonics GmbH, Bremen, Germany).

### 3.3. Modification of the HA–CT Conjugates with Suc-B12

Suc-B12 (25 mg, 0.018 mM) was dissolved in 2.5 mL DMSO, then 1.5-times molar amounts of EDC and NHS were added and stirred for 30 min for carbodiimide activation of the COOH groups. A 10 mL aqueous solution of HA–CT (55 mg, 0.18 mM) was added to the mixture and stirred overnight. The resulting product (HA–CT–B12) was dialyzed against distilled water for 5 days (until the pink color of the dialysis medium disappeared), and was then lyophilized.

The presence of vitamin B12 in the HA–CT–B12 conjugate was confirmed by FTIR using a Vertex 70 IR Fourier spectrometer (Bruker Optics, Ettlingen, Germany) equipped with a ZnSe-attenuated total reflectance accessory (PIKE Technologies, Fitchburg, WI, USA). A correction was applied to the attenuated reflectance spectra to account for the depth of penetration of the irradiation as a function of wavelength.

The conjugation efficiency was determined spectrophotometrically with a UV-1700 PharmaSpec spectrophotometer (Shimadzu, Kyoto, Japan) at 360 nm using a calibration with B12.

The CT content (μg/mg) in the HA–CT–B12 conjugate was calculated from the CT content in the HA–CT conjugate (367 μg/mg) determined by ^1^H NMR spectroscopy [7], and the B12 content in the HA–CT–B12 conjugate (95 μg/mg) was determined by UV-VIS spectrophotometry, as described above.

### 3.4. Size and Surface Morphology of the HA–CT–B12 Conjugates

The hydrodynamic radii and ζ-potential of HA–CT–B12 were measured using a Photocor Compact-Z device (Photocor Ltd., Moscow, Russia) with a 659.7 nm He–Ne laser at 25 mV power and a detection angle of 90°.

The morphology of HA–CT–B12 conjugates was analyzed by SEM using a Tescan Mira 3 instrument (Tescan, Brno, Czech Republic). For this purpose, the nanosuspension of HA–CT–B12 conjugates was centrifuged (15 min, 5000 rpm) to separate aggregates, and the obtained sample was placed on double-sided carbon tape and dried in a vacuum for 48 h; then, SEM images were obtained in the secondary electron mode at an accelerating voltage of 20 kV and an operating electric current of 542 pA; the distance between the sample and the detector was 6–7 mm.

### 3.5. The pH Stability Studies and In Vitro CT-Release Profile

To study the stability of the conjugates at the pHs of different parts of the GIT (stomach, duodenum, and ileum), 1 mg of the sample was placed in a Vivaspin^®^ Turbo 4 centrifugal concentrator (MWCO 10,000), and 1 mL of 0.1 M HCl at pH 1 (gastric conditions) was added. The sample was incubated at 37 °C, and after 2 h, the medium was completely ultracentrifuged at 4500 rpm and replaced with 1 mL phosphate buffer at pH 6.8 (pH of duodenal medium). The sample was incubated at 37 °C, and after 2 h, the medium was completely ultracentrifuged at 4500 rpm and replaced with 1 mL of PBS at pH 7.4 (pH of the ileum, the main site of vitamin B12 absorption, as well as blood pH). The sample was incubated for 10 h. At regular intervals, the medium was completely centrifuged at 4500 rpm and replaced with 1 mL of fresh PBS [7,24].

To determine the release profile of CT under conditions simulating the inflammatory site environment, 1 mg of the conjugate was dissolved in 1 mL of phosphate buffer at pH 5.2 (inflammatory site pH) and was incubated at 37 °C. At regular intervals, 1 mL of the medium was ultracentrifuged at 4500 rpm using a Vivaspin^®^ Turbo 4 centrifugal concentrator (10,000 MWCO). The CT content in the filtrates was determined by LC–MS, as previously reported [7,24].

### 3.6. Antimicrobial Activity

The antimicrobial activity of HA–CT–B12 was tested using the microtiter broth dilution method, as previously reported [7,24]. *P. aeruginosa* ATCC 27853 (Museum of Microbiological Cultures, State Research Institute of Highly Pure Biopreparations, St. Petersburg, Russia) was used as the experimental microorganism.

Briefly, HA–CT–B12 conjugate stock solutions were prepared by diluting samples in Mueller–Hinton broth to a maximum 2-fold concentration equivalent to CT. Serial dilutions of CT (64 to 0.25 μg/mL) were then plated on Luria-Bertani agar culture plates. The antimicrobial efficacy of the HA–CT–B12 conjugates was confirmed by the absence of *P. aeruginosa* growth.

*P. aeruginosa* suspension was serially diluted 1:100 in Müller–Hinton broth to obtain a concentration of approximately 1 × 10^7^ CFU/mL. Then, 125 µL of the inoculum was added to the wells of the culture plate containing HA–CT–B12 solutions in Müller–Hinton broth. The plate also contained positive controls—100% growth (bacteria only), sterility control (Mueller–Hinton broth only), and HA+B12 at equivalent concentrations (blank). The plate was incubated for 24 h at 37 °C and the optical density (OD) was measured at 630 nm using an ELx808™ Absorbance Microplate Reader (BioTek Instruments, Winooski, VT, USA). Relative bacterial growth (%) was calculated as the ratio of OD630 at each concentration of the test samples to OD630 in the control (0 μg/mL). Each sample was tested in triplicate in three independent replicates (*n* = 9).

### 3.7. Cytotoxicity Study

Cytotoxicity assays of the HA–CT–B12 were performed according to a previously described method [7,24] using a human embryonic kidney cell line (HEK 293) and a human glioblastoma cell line (T 98G). Briefly, the cell lines used were cultured at 37 °C in a 5% CO_2_ atmosphere in EMEM culture medium (Eagle’s minimal essential medium; Gibco, Billings, MT, USA) containing 1% essential amino acids, 10% (*v*/*v*) thermally inactivated fetal bovine serum (FBS; HyClone Laboratories, Logan, UT, USA), 1% L-glutamine, 50 U/mL penicillin, and 50 µg/mL streptomycin. For cytotoxicity studies, 5.0 × 10^3^ cells/100 μL/well were seeded in 96-well plates and cultured for 24 h; then, 100 or 50 μL EMEM solutions of the test compounds (1 mg or 0.5 mg CT and equivalent amounts of HA–CT–B12 conjugate or HA+B12 mixture) were added and the cells were incubated for 72 h. The medium was then removed and 50 μL EMEM medium containing 0.1 mg/mL 3-(4,5-dimethylthiazol-2-yl)-2,5-diphenyltetrazolium bromide (MTT reagent) was added, followed by incubation at 37 °C for 2 h. Finally, formazan crystals formed by metabolically viable cells were dissolved in dimethyl sulfoxide, and the optical density was measured at 570 nm using a UV mini-1240 spectrophotometer (Shimadzu, Kyoto, Japan).

### 3.8. Caco-2 Cell Permeability Assay

The human colon adenocarcinoma cell line Caco-2 was obtained from the Russian Cell Culture Collection (Institute of Cytology, Russian Academy of Sciences, St. Petersburg, Russia). Caco-2 cells were cultured in DMEM medium supplemented with 10% FBS, L-glutamine, and penicillin/streptomycin in 75 cm^2^ cell culture flasks (Jet Biofil, Guangzhou, China) at 37 °C in a humidified atmosphere containing 5% CO_2_ in a CO_2_ incubator. When the cells reached 60–70% confluence, the cells were sub-cultured into new flasks (1:6 ratio).

Caco-2 cell permeability protocol: For the experiment to study permeability and changes in the barrier properties of the intestinal epithelium, cells (0.5 mL, 100,000 cells per well) were seeded on the apical side of the 1.0 μm pore diameter membrane (Corning Incorporated, Corning, NY, USA) in cell culture inserts for 24-well plates. A total of 1 mL of complete DMEM medium was added to the basolateral chamber. The medium in the apical and basolateral chambers was changed every other day. Within 7 days, the cells formed a confluent monolayer and then polarized over the next 14 days, with tight junctions and microvilli formation, i.e., the cells acquired the properties of enterocytes. On day 21 of the cultivation, the medium was removed and the cell monolayer was washed three times with PBS. The studied samples (1 mg CT, 28.5 mg vitamin B12, and 300 mg HA–CT–B12 conjugate) were dissolved in 5 mL PBS, and 0.5 mL of the obtained solutions were added to the apical chamber. A total of 1 mL of PBS was added to the basolateral chamber, and 1 mL of PBS was removed from the basolateral chamber every 30 min for 2 h. This was followed by the addition of 1 mL of fresh PBS to maintain the same volume. The integrity of the Caco-2 monolayer was checked by leakage of 4 kDa fluorescein isothiocyanate–dextran (FITC–dextran, Sigma Aldrich (St. Louis, MO, USA)). A freshly prepared solution containing 5 mg/mL 4-kDa FITC–dextran dissolved in PBS was added to the apical chamber for incubation at 37 °C for 2 h. Samples were collected from the bottom chamber and fluorescence intensity was measured using a fluorescence plate reader (excitation 492 nm; emission 520 nm). If the FITC–dextran apparent permeability (Papp) was less than 1 × 10^−6^ cm/s, cells were considered acceptable for further experiments.

Each sample was assayed in three independent series (*n* = 3). The content of vitamin B12 and the content of the HA–CT–B12 conjugate equivalent to vitamin B12 were determined spectrophotometrically at 360 nm using a calibration curve. In addition, it was confirmed that the HA–CT–B12 conjugates were chemically stable under the conditions of this experiment and did not release free vitamin B12 within 2 h (as determined by spectrophotometric analysis).

The Papp was calculated using the following equation [33]:

(1) $$\mathrm{Papp}=\frac{dQ}{dt}*\frac{1}{A*C_{0}}$$

where Papp is the apparent permeability coefficient (cm/s); $\frac{dQ}{dt}$ —is the permeation rate (µg/s); *A* is the monolayer area (0.3 sm^2^); and *C*_0_ is the concentration in the apical chamber at the initial moment of time (µg/mL).

## 4. Conclusions

Oral drug delivery is characterized by simplicity and patient convenience as well as improved safety compared to intravascular injection. Increasing the intestinal permeability of peptide antibiotics is a current and challenging task. This study included several key aspects to address this challenge. First, we developed a convenient synthetic method to modify CT- and HA-based conjugates with a specific targeting ligand (vitamin B12) to improve intestinal permeability. Second, we demonstrated the stability of the obtained conjugates under gastrointestinal conditions (at pH 1, 6.8, and 7.4), as well as the preservation of antimicrobial activity at the level of free CT (2 µg/mL), with a decrease in nephrotoxicity (by 30–40%). Third, we demonstrated that modification with B12 actually improved the intestinal permeability of CT; the Papp of HA–CT–B12 conjugates was 3.5 × 10^−6^ cm/s, which is significantly higher than the Papp of pure CT (0.04 × 10^−6^). Furthermore, the Papp value of 3.5 × 10^−6^ cm/s corresponds to an in vivo intestinal absorption of 50–100%. Thus, B12 modification of CT- and HA-based conjugates may be an effective strategy for the development of oral delivery systems with improved bioavailability and a reduced toxicity profile of the peptide antibiotic CT.

## Figures and Tables

**Table 1:** Composition of Suc-B12-10 and Suc-B12-100 samples.

Molecular Ion	*m*/*z*	Fraction (%)
Suc-B12-10		
B12 sodium	1377	22.4
Monosuccinyl B12 sodium	1477	74.1
Disuccinyl B12 sodium	1577	3.5
Suc-B12-100		
B12 disodium	1399	0.4
Monosuccinyl B12	1455	87.8
Disuccinyl B12	1555	11.8

**Table 2:** Characterization of the HA–CT–B12 conjugate.

Parameter	Value
CT content (μg/mg)	335
B12 content (μg/mg)	95 ± 4
D_h_ (nm)	98 ± 22 702 ± 248
ζ-potential (mV)	−25.4 ± 0.3

**Table 3:** HA–CT–B12 conjugate stability under simulated gastrointestinal conditions.

Conditions	Time (h)	CT Release (%)
0.1 М НСl (pH = 1)	2	<0.2
Phosphate buffer (pH = 6.8)	2	<0.1
PBS (pH = 7.4)	10	<0.1

## Data Availability

The data are contained within the article.
